# The Relationship Between the Gut Microbiome-Immune System-Brain Axis and Major Depressive Disorder

**DOI:** 10.3389/fneur.2021.721126

**Published:** 2021-09-28

**Authors:** Jane A. Foster, Glen B. Baker, Serdar M. Dursun

**Affiliations:** ^1^Department of Psychiatry and Behavioural Neurosciences, McMaster University, Hamilton, ON, Canada; ^2^Department of Psychiatry and Neuroscience and Mental Health Institute, University of Alberta, Edmonton, AB, Canada

**Keywords:** axis, depression, gut microbiome, immune system, brain, probiotics, prebiotics, vagus nerve

## Abstract

Major depressive disorder (MDD) is a prominent cause of disability worldwide. Current antidepressant drugs produce full remission in only about one-third of MDD patients and there are no biomarkers to guide physicians in selecting the best treatment for individuals. There is an urgency to learn more about the etiology of MDD and to identify new targets that will lead to improved therapy and hopefully aid in predicting and preventing MDD. There has been extensive interest in the roles of the immune system and the gut microbiome in MDD and in how these systems interact. Gut microbes can contribute to the nature of immune responses, and a chronic inflammatory state may lead to increased responsiveness to stress and to development of MDD. The gut microbiome-immune system-brain axis is bidirectional, is sensitive to stress and is important in development of stress-related disorders such as MDD. Communication between the gut and brain involves the enteric nervous system (ENS), the autonomic nervous system (ANS), neuroendocrine signaling systems and the immune system, and all of these can interact with the gut microbiota. Preclinical studies and preliminary clinical investigations have reported improved mood with administration of probiotics and prebiotics, but large, carefully controlled clinical trials are now necessary to evaluate their effectiveness in treating MDD. The roles that several gut microbe-derived molecules such as neurotransmitters, short chain fatty acids and tryptophan play in MDD are reviewed briefly. Challenges and potential future directions associated with studying this important axis as it relates to MDD are discussed.

## Introduction

Major depressive disorder (MDD), one of the leading causes of disability worldwide (1, 2), is considered a stress-related disorder, and it has been proposed that stress resulting from negative life events, including those occurring early in a person's life, is a contributing factor to the development of MDD (3–5). The antidepressant drugs currently available provide full remission in only about 1/3 of patients, and with most of these drugs there is a 2–3 week delay before clinical improvement becomes obvious and they have some undesirable side effects which may interfere with patient compliance (6). The biogenic amine hypothesis of depression states that depression is due to a functional deficiency of 5-hydroxytryptamine (5-HT, serotonin) and/or norepinephrine at specific synapses in the brain, and most of the currently available antidepressants increase availability of these biogenic amines by inhibiting their reuptake into nerve terminals, inhibiting their metabolism and/or acting on their receptors. Although the biogenic amine hypothesis has been useful in drug development, it has become obvious in recent years that many other factors in addition to 5-HT and norepinephrine are important in the etiology and therapy of MDD (6).

Current treatments often do not address adequately the needs of individuals who do not respond to front line therapy, and the existing approach to care often fails to provide individuals with a path to wellness. The economic burden of depression is significant, such that there is an immediate need to consider novel ways to understand the heterogeneity of clinical phenotypes in depression. Attention to identifying biomarkers to advance precision medicine approaches to guide clinicians in choosing the best treatment for individuals has moved to the forefront of psychiatric research. This requires a better understanding of the etiology of MDD and to have new targets for future development of more effective antidepressant therapies. This need has led to exciting studies in recent years on the involvement of the immune system (7–9) and the gut microbiome (5, 10, 11) in the etiology and treatment of MDD. In this paper, we will give a brief overview of each of these areas and give an update on them with regard to their interactions in MDD and how this may influence new drug discovery as well as microbiome-targeted approaches to treatment.

## The Immune Sytem and MDD

For many years, it has been felt that the immune system plays an important role in MDD; for example, some symptoms of depression accompany infections in humans, and people suffering from autoimmune disorders often have comorbid depression (5). It has also been reported that treatment of hepatitis C with proinflammatory agents like interferon-alpha (IFN-α) results in depressive symptoms in many patients (12). Since the 1990s, there have been extensive studies indicating that chronic inflammation and hypersecretion of molecules like the proinflammatory cytokines play a role in the etiology of depression (7, 13, 14), although, in contrast, suppression of immune responses have also been reported in some depressed patients (15). Inflammation can result in disruption of the blood-brain barrier (BBB), cellular and structural changes in the central nervous system, induction of glutamate release from microglia and impairment of long-term potentiation (9). Several antidepressants have been reported to have anti-inflammatory effects and/or to reduce levels of proinflammatory cytokines in a variety of animal models and macrophages (9, 16–20), although not all studies agree (21, 22). Changes in levels of several immune markers have been reported to be helpful in predicting efficacy of antidepressants (review: 9). Del Grande da Silva et al. (23) have reported that reduction of depressive symptoms by using cognitive behavioral therapy (CBT) also results in a modulation of levels of the proinflammatory cytokines interleukin-6 (IL-6) and tumor necrosis factor-α (TNF-α). It has been observed in several studies that classic proinflammatory cytokines such IL-1β, IL-6, and TNF-α are negative modulators of hippocampal neurogenesis (24–26). Cytokines have also been reported to have significant effects on synthesis, release and reuptake of several neurotransmitters such as serotonin, dopamine and glutamate (27). The levels of IL-1β in the hippocampus and its effect on hippocampal neurogenesis have been reported to be reduced by antidepressants (28–30). Notably, researchers in psychiatry and behavioral neuroscience are increasingly recognizing the importance of the immune system in behavior (31), such that studies on interaction of stress and the immune system may result in novel therapeutic targets for treatment of depression in patients in whom inflammation plays a major role (9, 32). Expanding the scope of stress-immune interactions, recent findings have revealed a new player, namely the gut microbiome, which appears to be a key regulator of stress and inflammation (5). Gut microbiota are essential to immune system development and immune function. The gut microbiome is a critical regulator involved in the tuning of the mucosal immune system (33). Intestinal epithelial cells are at the interface between the microbiota and the mucosal immune system and able to influence the composition of the microbiota, and research has provided evidence for a reciprocal relationship, with commensal microbes involved in proper development and function of immune cells and these cells in return influencing the habitat and diversity of the microbiota (34). Given the interactions between the gut microbiome and the immune system (35–39) and the proposed involvement of the immune system in MDD, it is important to consider more broadly the microbiota-immune-brain axis in MDD.

## The Gut-Brain Axis and MDD

Interest in the gut-brain axis was stimulated greatly by investigations in germ-free mice, lacking all microbes, and subsequent studies suggested that the gut microbiome could influence neurodevelopment, neuroplasticity, neurotransmsitter systems, neurogenesis and several behavioral phenotypes (40–42). Gut microbiota contribute to healthy metabolism and play a critical role in the normal development of the immune, endocrine, and nervous systems (35–39). Based on many studies in the past 15 years, we know that the link between stress and microbiota is bidirectional – stress can influence the microbiota and the microbiome can influence the impact of stress (43–45). For example, exposure to various types of stress has been reported to alter the microbiota profile, and alteration of the gut microbiota can affect responses to stress, anxiety-like behavior and, as described below, activation of the hypothalamic-pituitary-adrenal (HPA) axis [reviews: (40, 44)]. It has been shown that the intestinal microbiota are required for some of the stressor-induced changes in inflammation (45, 46). Stress can increase intestinal permeability, allowing bacteria to move across the intestinal mucosa to directly act on immune cells as well as neuronal cells of the enteric nervous system (47).

Microorganisms within the human gut include bacteria, viruses, fungi, archaea, and eukaryotes, weighing on average 2 kg in the human adult. It has been suggested that the human gastrointestinal tract (GI) is comprised of over 100 trillion viable bacteria, making up just more than 50% of the cells within the body (48, 49). The focus in this minireview is on gut bacteria. The gut microbiome is important for the development and function of the HPA axis, which mediates stress responses and is known to be hyperactive in many patients with MDD (50). The HPA axis can be modified markedly by the composition of the gut microbiome (51, 52), and an overactive HPA axis has been reported in both germ-free mice and rats (52, 53). These latter authors have also reported differences in turnover of norepinephrine, serotonin and dopamine in several brain regions of germ-free male rats compared to controls (53). It has been reported that germ-free mice have an under-developed mucosal immmune sytem, a less effective immune reponse to infection and endotoxin challenge, fewer regulatory T cells (Treqs) with reduced antiinflammatory capacity, and global defects in their microglia (review: 54).

Although the focus of the present minireview is on depression, alterations in the gut microbiota composition have been reported in a wide variety of psychiatric and neurologic disorders (50, 54–56). Over 1,000 distinct bacterial species inhabit the human gut, with the most prominent phyla being *Firmicutes* and *Bacteroides* (57, 58). It has been reported that compared to healthy controls, MDD patients show increased *Bacteroidetes, Protobacteria* and *Actinobacteria* and less *Firmicutes* *(**59*, *60**)*. With regard to genus changes, MDD patients have been reported to have increased *Enterobacteriaceae* and *Allistipes* and less *Faecalibacterium*, the last of these inversely correlated with severity of depression (59). A recent large population study reported an association between the microbiome and quality of life and depression (61); abundances of *Faecalibacterium* and *Coprococcus* bacteria were associated with higher quality of life, and reductions of *Coprococcus* and *Dialister* spp. were linked to depression, an observation that was validated in a second cohort (61). In a study in mice, Lukic et al. (62) investigated chronic admnistration of 5 different antidepressants (fluoxetine, escitalopram, venlafaxine, duloxetine and desipramine) and found that the antidepressants reduced abundance of *Ruminococcus* and *Adlercreutizia*. Further studies with duloxetine and *Ruminococcus flavefaciens* found that this microbe reduced the antidepressant-like properties of duloxetine. The authors also found that administration of *Ruminococcus flavefaciens* upregulated genes involved in mitochondrial oxidative phosphorylation and downregulated genes involved in synaptic signaling and neurogenesis as well as reducing levels of 5-HT and norepinephrine in the prefrontal cortex (62). In a study in which metagenomics sequencing of fecal samples from a population cohort and two gastrointestinal disease cohorts were investigated, selective serotonin reuptake inhibitor (SSRI) antidepressants increased abundance of *Eubacterium ramulus* in patients with irritable bowel syndrome (IBS), and this increased abundance was observed mainly in users of paroxetine (63). In a review on the interactions between a variety of drugs and the gut microbiome in humans, Weersma et al. (64) reported that a number of commonly used drugs, including SSRI antidpressants, influenced gut microbiome composition and functions and reported that there can be a birectional interaction with various drugs, with gut microbes altering bioavailabilty, bioactivity and/or toxicity by metabolizing drugs enzymatically.

## The Gut Microbiome-Immune System-Brain Axis

**Figure 1:**
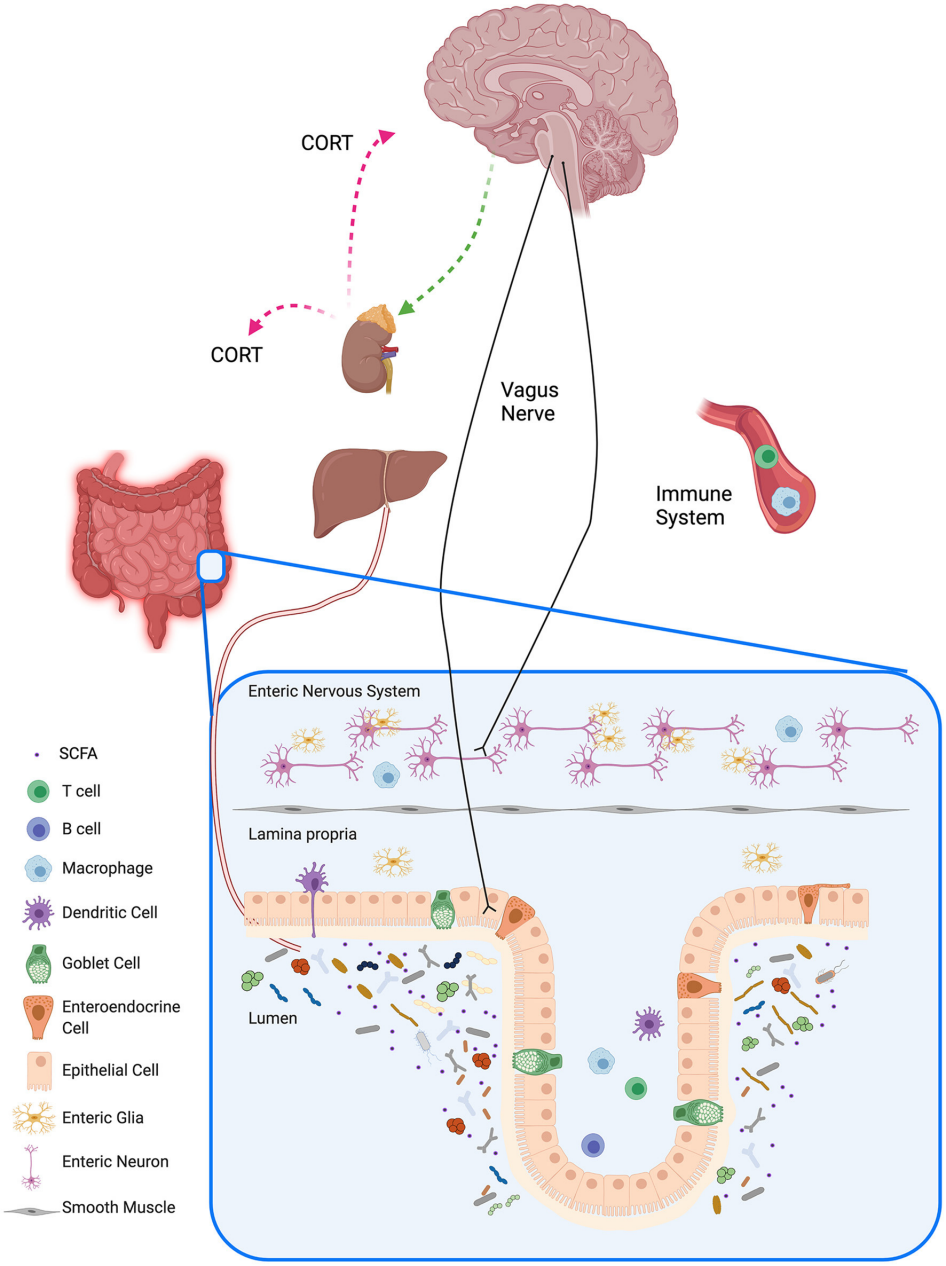
Gut-brain Axis. The gut-brain axis is the bidirectional communication between the gut and the brain, and its communication is associated by different pathways with the autonomic nervous system, the enteric nervous system, the neuroendocrine system, and the immune system. Trillions of gut microbes and their metabolites reside in the lumen of the gastrointestinal tract and influence the local mucosal system (visualized in the inset for the barrier in the colon). Signaling pathways between the gut microbiota and the brain include neural pathways such as the vagus nerve, immune pathways, and humoral pathways. Gut microbiota influence host metabolism through local interactions, peripheral systems, and bidirectional communication with the liver. Schematic created with BioRender.com.

Much of the previous research on the involvement of the gut microbiome and the immune system on brain function and MDD has focused on these systems individually, but there is now considerable interest in studying how the bidirectional communication between the gut microbiome, the immune system and the brain is integrated and related to MDD (Figure 1). Many preclinical and clinical studies have focused on the importance of the gut-brain axis and the various routes of communication along the axis; research to date suggests that alterations in microbiota can modualte the following sytems in the brain: plasticity-related [e.g. through effects on brain-derived neurotrophic factor (BDNF)], serontonergic and GABAergic systems [review: (46)]. Gut microbiomes can also produce a number of active metabolites that can affect brain function, although in some cases the precise mechanism by which the effects occur are not yet clearly understood (see the following section on microbiome-derived molecules). Butler et al. have provided a detailed table summarizing pathways involved in the axis and their their relevance to the pathogenesis of psychiatric disorders (50). The gut microbiota can interface with the peripheral nervous system, e.g. with the vagus nerve, to send signals to the central nervous system, and in the other direction the central nervous system can send signals to the gut, modulating the composition and function of the microbiota (11). The mucosal, peripheral and central immune systems are in constant communication with the gut microbiota (65), and the endocrine system also communicates bidirectionally with the gut microbiota, mainly through the HPA axis (66). The gut epithelium is an intricate barrier allowing nutrients, electrolytes and water inside the lumen to be absorbed into the circulation and to prevent pathogens and toxins from entering (67, 68). During periods of chronic stress, the gut epithelial layer can become more permeable, leading to increased movement of endotoxins from inside the gut to the outside, resulting in a low-grade inflammation which is characteristic of a number of disorders, including MDD. Interestingly, it has been reported that treatment with probiotics can correct some of the changes related to increased gut permeability (47, 69). Sylvia and Demas (68) have provided a comprehensive table of preclinical studies on interactions among the microbiome, the immune and endocrine systems and social and affective behaviors.

## Microbiome-Derived Molecules

The following molecules which may affect brain function are formed by the gut microbiome: neurotransmitters [biogenic amines, acetylcholine and γ-aminobutyric acid (GABA)], short chain fatty acids (SCFAs), indoles (tryptophan and its metabolites), bile acids, choline and metabolites, lactate and vitamins (70). It has been proposed that these molecules may affect depressive behavior through direct and indirect mechanisms, including: direct stimulation of receptors in the brain; stimulation of neural, endocrine and immune mediators in the periphery; and epigenetic regulation (histone acetylation and DNA methylation) (70). Many of the prominent neurotransmitters present in the brain (e.g. norepinephrine, 5-HT, acetylcholine, GABA) are also present in the gut, and absence of the gut microbiome (e.g. in germ-free rodents) results in a reduction of intestinal levels of norepinephrine (71), 5-HT (72) and GABA (73). It is difficult to ascertain the exact mechanisms by which these neurotransmitters may affect the brain directly (74) because of difficulties passing the blood-brain barrier (BBB), but they may affect brain levels of the neurotransmitters by modulation of the immune system either directly or by affecting cytokine production by local stimulation of the vagus nerve (70, 75–79). Microbiota have a key role in regulating the metabolism of tryptophan, an essential amino acid that comes from the food we eat. Tryptophan is the precursor for serotonin, but the majority of tryptophan in the body is metabolized through the kynurenine pathway (metabolites include quinolinic acid and kynurenic acid, which have neurotoxic and neuroprotective properties, respectively), whereas only a small percentage of tryptophan is metabolized to serotonin (80–84). Tryptophan is metabolized to a wide variety of metabolites after initial catabolism by indoleamine-2.3-deoxygenase (IDO) and tryptophanase by the gut micorbiota, and several of these indoles can inhibit neuroinflammation (85). Specific to the serotonin pathway, tryptophan is able to cross the blood-brain barrier (BBB) where it participates in the synthesis of serotonin within the raphe nuclei in the brain stem (83, 84). Other microbiota-derived metabolites may also play a key role in depression (70). Short-chain fatty acids (SCFAs), e.g. acetate, butyrate and propionate, are produced in microbiota by anaerobic fermentation of predominantly indigestible carbohydrates. These SCFAs have wide-ranging physiological actions such as effects on energy regulation, lipohomeostatis, regulation of the intestinal barrier integrity, modulation of the BBB, BDNF expression, development and function of the immune system and modulation of epigenetics (70). In addition, cross-talk between microbiota is influenced by SCFAs and by gene expression related to the production of SCFAS. SCFAs have shown antidepressant-like actions in several studies in rodents (70), although other studies have not observed abnormal levels of butyrate in humans with MDD (86, 87) or in animal models of depression (88). The physiological effects of lactate, bile acids, choline metabolites and the vitamin folic acid produced by the gut microbiota and their possible relationship to MDD are reviewed by Caspani et al. (70).

## Probiotics and Prebiotics

Probiotics are consumable microbiomes meant to be adminstered to promote a healthier microbiome. Prebiotics, which include complex carbohydrates and plant polysaccharides that can be catabolized by gut microbiota, can affect several metabolic pathways in the body through their metabolic by-products, SCFAs (89). Probiotics and prebiotics have been reported to dampen the stress response, decrease intestinal permeability, decrease inflammation, and improve the symptoms of depression (54). The term psychobiotics refers to probiotics and prebiotics that signficantly affect the brain and behavior, with an emphasis on mental health aspects (11, 90). Several researchers have reported that probiotics and prebiotics have anti-anxiety- and/or antidepressant-like effects in rodent models (46, 71, 78, 91, 92). Researchers have demonstrated a beneficial effect of probiotics and prebiotics on anxiety, depression and stress systems in healthy individuals and in clinical populations. Following administration of probiotics, benefits include improved mood, reduced depression and anxiety scores, reduced stress hormones, improved neurocognitive performance, reduced stress-induced memory changes, and reduced inflammation (93–106). Interpersonal variability in microbiota composition is high, but each person has a distinct microbial profile, and there are similarties in the relative abundance and distribution of bacterial species among healthy persons (55); therefore microbiome-based biomarkers may help subtype clinical populations into more homogenous groups to predict best treatment, including with microbiota-targeted therapies such as probiotics.

## Challenges and Current Research Gaps Related to Studying The Microbiome-Immune System-Brain Axis in Depression

MDD is a heterogenous disorder, with patients diagnosed as having MDD by current criteria (e.g. using DSM-5) potentially having quite different groups of symptoms from each other. The situation is made even more challenging since comorbities are often present, with anxiety disorders and gastrointestinal disorders common. It is hoped that future research will find associations of specific gut microbes with specific features of MDD, resulting in application of gut microbes as biomarkers for following the course of improvement of MDD with treatment. Hopefully, this research will also provide information about gut microbiome markers that will be useful in predicting the likelihood of an individual developing MDD and possibly using this information to provide a healthier gut microbiome, i.e. promoting wellness using preventative measures. As with many neuropsychiatric disorders, the translation of findings in animal models to the clinical situation could turn out to be disappointing; more clinical information on the gut microbiome-immune system-brain axis is and its modification by drugs, psychotherapy and pro- and prebiotics is required. Although there is now a reasonable body of preclinical evidence on the effects of current antidepressant therapies and of pro- and prebiotics on the immune system, gut microbe composition and metabolites formed by the gut microbiota, this research should be conducted, where possible, in longitudinal clinical trials.

There are also still gaps in our knowledge of how metabolites formed by gut microbes act exactly to affect the immune system and brain function. There are also some problems with how the gut microbe composition is currently measured. Although differences between MDD patients and healthy controls with regard to this composition have been reported (42), it is important to be able to identify key taxa and to associate them with physiological changes in patients. Analyical tools used most frequently to date, such as 16S rRNA gene sequencing, do not provide specificity for taxa measurements, and there is now increasing focus on using approaches such as shotgun metagenomics for taxa measurements and also employing metabolomics to measure levels of the metabolites of the microbes and/or the host (42).

## Future Directions

Future studies should relate changes at specific time points in the microbiome composition and the metabolome to changes in MDD symptoms produced by various types of treatment (10). Two very important areas of research that must be investigated in more detail in the future are sex-dependent differences in composition of the gut microbiome and increasing our understanding of the influence of early life exposure to stress and inflammation on the gut microbiome (46, 55). Large longitudinal clinical studies should be done to further investigate the effects of stress on the gut microbiome to determine if this may be important in an individual's susceptibilty to developing depression (5). In order to advance the translation of microbiome research to the clinic, it will be necessary to use techniques such as shotgun metagenomics sequencing and metabolomics (42). At the prelinical level, it is important to investigate more extensively the involvement of the gut microbiome in adult hippocampal neurogenesis, given the apparent importance of this neurogenesis in antidepressant action (5). While microbiome-based approaches using probiotics and prebiotics have received attention, more studies need to be conducted in future on fecal microbiota transplant (FMT) (104), a technique for repopulating the gut microbiome. Chinna Meyyappan et al. (107) did a systematic review on the effect of FMT on symptoms of psychiatric disorders. They investigatead 8 studies that were entirely clinical, 9 that were preclinical with human donors and 11 that were entirely preclinical. They reported that all studies found decreased depressive- and anxiety-like symptoms when healthy microbiota were transplanted and that transplantation of microbiota from psychiatrically ill donors to healthy subjects resulted in transmission of the depressive- and anxiety-like symptoms. These authors recommended that future research in this area should include larger sample sizes and stronger designs in order to determine the efficacy and safety of FMT (107). For a recent review of possible risks involved with FMT, see (108). In addition, the field of nutritional psychiatry is expanding and the potential for diet and lifestyle factors in treating and preventing depression must be considered (41, 109).

## Conclusion

Despite some controversies and some as yet unanswered questions about integration and about composition of the gut microbiome, the axis involving the gut, the immune system and brain function has exctiting implications for furthering our understanding of the etiology of MDD and for its treatment. As summarized by Foster (42), important reasons to consider the microbiome in clinical psychiatry are identification of biomarkers related to biological differences that will improve ability to match indiviuals with the best treatments for them; identification of at-risk individuals for whom early intervention could be made available; identification of novel targets for drug development; and development of microbiome-targeted therapies such as diet, prebioltics and probiotics.

## Author Contributions

All authors worked on the literature search. JF provided Figure 1 through her paid license with BioRender. GB wrote the first draft of the manuscript and all authors worked on the editing. The submitted version of the manuscript has been approved by all the authors.

## Funding

GB has a TRIP research allowance (TRP-GB) from the Faculty of Medicine & Dentistry at the University of Alberta. JF has funding from CIHR, NSERC RGPIN-312435-12 (JF), and the Ontario Brain Institute.

## Conflict of Interest

The authors declare that the research was conducted in the absence of any commercial or financial relationships that could be construed as a potential conflict of interest.

## Publisher's Note

All claims expressed in this article are solely those of the authors and do not necessarily represent those of their affiliated organizations, or those of the publisher, the editors and the reviewers. Any product that may be evaluated in this article, or claim that may be made by its manufacturer, is not guaranteed or endorsed by the publisher.
